# Factors associated with anaemia in kidney transplant recipients in the first year after transplantation: a cross-sectional study

**DOI:** 10.1186/s12882-018-1054-7

**Published:** 2018-10-05

**Authors:** Andy K.H. Lim, Arushi Kansal, John Kanellis

**Affiliations:** 1 Department of Nephrology, Monash Health, Clayton, Victoria 3168 Australia; 2 Department of Medicine, Monash University, Clayton, Victoria 3168 Australia

**Keywords:** Anaemia, Kidney transplantation, Haemoglobin, Iron-deficiency, Haematinics, Blood transfusion

## Abstract

**Background:**

Anaemia after kidney transplantation may reduce quality of life, graft or patient survival. We aimed to determine the prevalence and risk factors for anaemia in the initial 12 months after transplantation.

**Methods:**

We conducted a cross-sectional study at 6 and 12 months after transplantation. Anaemia was defined by World Health Organization criteria taking into consideration erythropoietin use. Logistic regression was used to determine the association between demographic, clinical and pharmacological risk factors for the main outcome of moderate-severe anaemia.

**Results:**

A total of 336 transplant recipients were included and the prevalence of moderate-severe anaemia was 27.4% at 6 months and 15.2% at 12 months. Lower kidney function, female gender, transferrin saturation below 10% and proteinuria were associated with moderate-severe anaemia at both time points. Recent intravenous immunoglobulin treatment was associated with anaemia at 6 months. Recent infection and acute rejection were also associated with anaemia 12 months. Around 20% of patients had at least one blood transfusion but they were uncommon beyond 3 months.

**Conclusions:**

Anaemia remains highly prevalent requiring treatment with erythropoietin and transfusions. Most identifiable risk factors relate to clinical problems rather than pharmacological management, while markers of iron-deficiency remain difficult to interpret in this setting.

**Electronic supplementary material:**

The online version of this article (10.1186/s12882-018-1054-7) contains supplementary material, which is available to authorized users.

## Background

Post-transplant anaemia affects 10–40% of kidney transplant recipients in the first 12 months. The prevalence partly depends on the definition of anaemia and timing post-transplant [1]. Transplant patients have more anaemia than the GFR-matched general population, suggesting that the transplantation process itself may contribute to anaemia [2].

Anaemia requiring transfusions is a risk factor for immunological sensitisation, which may affect future re-transplantation. Post-transplant anaemia is also associated with left ventricular hypertrophy, reduced systolic function and long-term mortality [3, 4]. In the French DIVAT study, anaemia at 12 months based on World Health Organization (WHO) criteria was associated with reduced patient survival in those with chronic kidney disease (CKD) stages 1–3 [5]. Anaemia may also reduce graft survival [6–9], quality of life and affect mental health [10, 11].

Early anaemia is often due to surgical factors, haemodilution and withdrawal of previous erythropoiesis-stimulating agents (ESA). Most anaemia resolves by 3–6 months with restoration of erythropoietin levels. Anaemia after this time frame is particularly relevant as the potential causes are less obvious. The prevalence of anaemia has also changed with the evolving immunosuppressive practices and use of co-administered medications (era-effect). Thus, evaluation of anaemia requires consideration of both clinical and pharmacological factors, and extrapolating from the general CKD population is not necessarily valid.

We aimed to determine the prevalence of anaemia and haematinic deficiency at 6 and 12 months in a contemporary kidney transplant cohort, and to determine the risk factors associated with anaemia. We focussed on moderate-severe anaemia, as patients with mild anaemia are not likely to be candidates for ESA intervention and the long-term consequences of mild anaemia may be less significant.

## Methods

### Study design and patients

This was a cross-sectional study of all adult (> 18 years) kidney transplant recipients including combined kidney-pancreas transplants from a single centre (Monash Health). The time points examined were at 6 and 12 months post-transplantation. The study period included transplants performed from 1 Jan 2011 to 31 Dec 2015. Patients were excluded from the study if they were deceased or returned to dialysis within 12 months post-transplantation. Patients with inadequate clinical data were also excluded.

### Data collection

Clinical information was obtained from electronic medical records, including demographics (age, sex, diabetes, polycystic kidney disease [PKD], vasculitis and gastrointestinal bleeding risk) and transplantation details (donor type, delayed graft function, combined pancreas-kidney).

Information on clinical progress recorded included: *Recent (within the last 3 months)* episode of recognised bleeding, acute rejection, cytomegalovirus viraemia or nephropathy, BK virus viraemia or nephropathy. *Recent (within the last 4 weeks)* clinically evident systemic infection determined by history, examination and/or laboratory or imaging tests; for example, urinary or respiratory infections. We did not collect qualitative data on symptoms related to anaemia.

Information on medications (immunosuppressant, ESA, proton-pump inhibitors, anticoagulants, anti-platelets, renin-angiotensin system inhibitor, valganciclovir, trimethoprim-sulfamethoxazole, iron supplementation or infusion, vitamin supplementation or injections), treatments for rejection (plasma exchange, intravenous immunoglobulin [IVIG]) and episodes of blood transfusions were also extracted.

Laboratory data was obtained from routine follow up tests per transplant protocols. This included haematinics, parathyroid hormone (PTH) and urinary protein excretion at 6 and 12 months post-transplantation. Laboratory results up to 6 weeks before or after the study time points were considered acceptable for this cross-sectional design. Therefore, missing laboratory data could be due to true missing results or tests performed outside the accepted time frame.

The transplant physicians used their discretion to investigate potential causes of anaemia. They may have organised endoscopy or specialist haematological assessment. We did not collect data on any additional anaemia work-up beyond that routinely collected per protocol.

### Definitions

Anaemia was defined by gender-specific WHO criteria: mild anaemia in male 110–129 g/L, female 110–119 g/L; moderate anaemia < 110 g/L, severe anaemia < 80 g/L. A haemoglobin of < 110 g/L defines moderate-severe anaemia for both genders. Patients requiring ESAs to maintain their haemoglobin levels were considered to have moderate-severe anaemia as these patients had a haemoglobin level < 100 g/L to qualify for ESA treatment.

B12 deficiency was defined as a serum level < 140 pmol/L or receiving B12 injections initiated within the last 3 months due to a documented deficiency. Low ferritin was defined as a level < 20 μg/L. Low transferrin saturation was defined as < 15%. Folate deficiency was defined as a serum folate < 10 nmol/L or red cell folate < 800 nmol/L. Serum PTH level is normally between 1.0 and 7.0 pmol/L. We analysed proteinuria as a categorical variable because a 24-h urine collection result was not available for all patients. We defined a 24-h urine protein excretion greater than 0.1 g/day or a spot urine protein-creatinine ratio greater than 0.03 g/mmol, as a positive result. Urine protein-creatinine ratios were also grouped into three ordinal levels: (1) ≤0.03 g/mmol, (2) > 0.03 to ≤0.1 g/mmol, (3) > 0.1 g/mmol.

### Statistical analysis

All analyses were performed with STATA, version 15 (StataCorp, TX USA). To compare continuous variables at 6 and 12 months, a paired t-test or Wilcoxon signed-rank test was used depending on the distribution of the variables. To compare paired proportions for dichotomous variables, Mc Nemar’s test was used. Logistic regression was used to analyse the association between the clinical and pharmacological predictors and the main binary outcome of anaemia for each time point. Variables with *P* < 0.10 in univariable analysis were included in a baseline multivariable model and a backward-elimination method was used to determine a final multivariable model. In the final model, multiple imputation was performed for missing transferrin saturation data, using a linear regression imputation method (imputed datasets, m = 50). The variables used in the imputation model were: age, gender, haemoglobin, haematocrit, mean corpuscular volume, white cell count, recent infection, recent rejection and proteinuria. We used orthogonal polynomial contrasts of the marginal predictions from the multivariable models to test for trend in the association between urine protein-creatine ratio levels and anaemia. The test for trend was conducted on five individual imputed datasets at both 6 and 12 months, and the conservative *P*-values were reported. A *P*-value less than 0.05 was considered statistically significant (or *P* < 0.01 when testing for interaction).

## Results

### Patient demographics

**Fig. 1:**
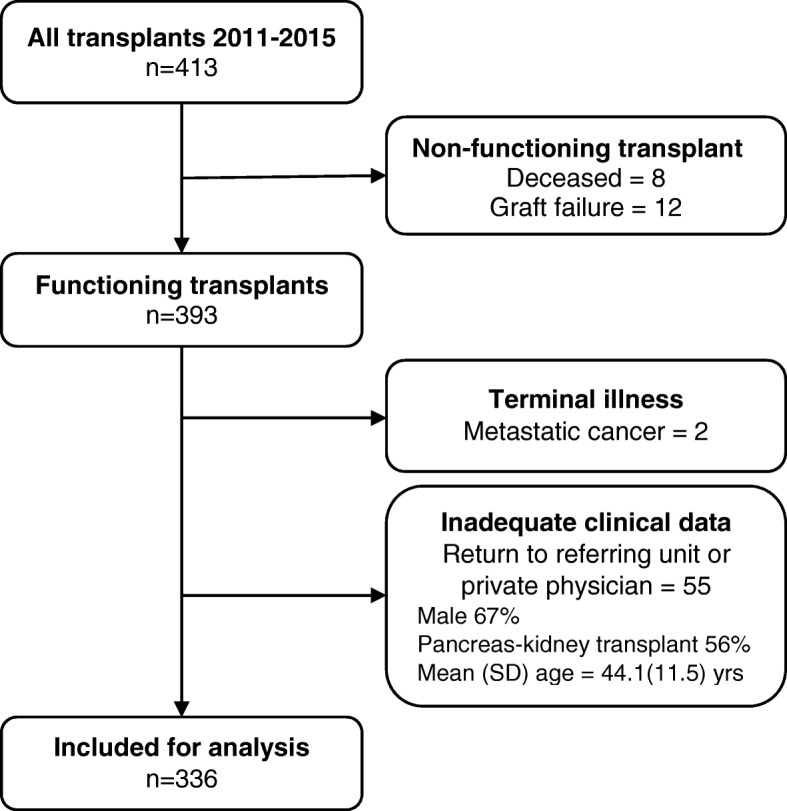
Study flowchart. Flowchart showing number of kidney transplant recipients in the database and reasons for exclusion from the final analysis

A total of 413 patients were in the database from 1 Jan 2011 to 31 Dec 2015. Of these, 336 patients were suitable for analysis (Fig. 1) and the demographics of the included patients are shown in Table 1. Most of exclusions were due to the lack of follow-up data. There was a slight preponderance of males. There were only 63/336 (18.8%) cardiac death donors but these accounted for 68.3% of all cases of delayed graft function (post-transplant dialysis). There were 32.7% pre-existing diabetics and 5.1% newly diagnosed post-transplant. Known upper gastrointestinal disorders were more common than lower gastrointestinal disorders as risk factors for gastrointestinal bleeding.

**Table 1:** Characteristics of included patients (*n* = 336)

Characteristic	n (%)
Mean (± s.d.) age *years*^a^	51.3 ± 12.9
Male sex	218 (64.9)
Donor type:	
Brain death	176 (52.4)
Cardiac death	63 (18.7)
Living	82 (24.4)
ABO incompatible	15 (4.5)
Delayed graft function^b^	83 (24.7)
Pancreas-kidney transplant	23 (6.9)
Polycystic kidney disease	37 (11.0)
Diabetes:	
Type 1	39 (11.6)
Type 2	71 (21.1)
New-onset after transplant	17 (5.1)
Gastrointestinal bleeding risk:	
Upper gastrointestinal^c^	38 (11.3)
Lower gastrointestinal^d^	21 (6.3)
Vasculitis	11 (3.3)

^a^age at 6-month follow-up

^b^needed at least one dialysis treatment

^c^gastro-oesophageal reflux, oesophagitis, gastritis or ulceration

^d^polyps, angiodysplasia, diverticular disease or haemorrhoidal

### Clinical characteristics

Recognised bleeding (within prior 3 months) was infrequent at both 6 and 12 months (5.1 and 3.3%, respectively). Episodes of biopsy proven rejection (within prior 3 months) were common (14.0% at 6 months, 11.3% at 12 months). Isolated cellular rejection was less common (< 2%), with the majority of biopsies showing some element of antibody-mediated rejection. The proportion of patients with a recent infection (within prior 4 weeks) was higher at 6 months (20.8%) than 12 months (13.7%), which was statistically significant (difference 7.1%, 95%CI 1.5–13.8%; *X*^2^ = 6.70, df = 1, *P* = 0.010). A recent cytomegalovirus viraemia or infection (within prior 3 months) was uncommon at both time points (3.9%). BK viraemia or infection (within prior 3 months) was more common (15.2% at 6 months, 14.3% at 12 months).

### Immunosuppression and medications

A comparison of immunosuppression and medication use for both time points is shown in Table 2. At 12 months, patients were generally on lower mycophenolate mofetil (MMF) doses and lower average prednisolone use compared to 6 months. Calcineurin-inhibitor use was no different with most patients on tacrolimus. As calcineurin-inhibitor doses were targeted to levels, the actual dosage carries little meaning and was not examined. Plasma exchange and IVIG use were less frequent at 12 months compared to 6 months. Our centre does not use anti-thymocyte globulin or rituximab for induction therapy. We use an interleukin-2 receptor antibody (basiliximab) for induction in high immunological risk patients. Basiliximab effects are not expected to last 6 months.

**Table 2:** Medications and immunosuppression (*n* = 336)

Medication/immunosuppression	6 months n (%)	12 months n (%)	*P* value
Mycophenolate dose < 1.5 g/day	70 (20.8)	107 (31.8)	< 0.001
Calcineurin inhibitor:			
Tacrolimus	329 (97.9)	325 (96.7)	0.13^b^
Cyclosporine	4 (1.2)	8 (2.4)	
None	3 (0.9)	3 (0.9)	
Sirolimus/everolimus	11 (3.3)	7 (2.1)	0.13^b^
Prednisolone^a^	6.9 (2.6)	5.3 (1.5)	< 0.001
Plasma exchange	18 (5.4)	8 (2.4)	0.041
Intravenous immunoglobulin	49 (14.6)	28 (8.3)	0.006
Proton pump inhibitor	301 (89.6)	287 (85.4)	0.006
Trimethoprim-sulfamethoxazole	301 (89.6)	256 (76.2)	< 0.001
Valganciclovir	180 (53.4)	51 (15.2)	< 0.001
Renin-angiotensin inhibitor	49 (14.6)	60 (17.9)	0.055
Anticoagulation			
None	239 (71.1)	238 (70.8)	1.00^b^
Aspirin	82 (24.4)	78 (23.2)	
Clopidogrel	2 (0.6)	2 (0.6)	
Dual antiplatelets	1 (0.3)	2 (0.6)	
Warfarin	8 (2.4)	10 (3.0)	
Novel anticoagulants	4 (1.2)	6 (1.8)	
Iron supplementation			
None	321 (95.5)	322 (95.8)	1.00^b^
Oral	7 (2.1)	8 (2.4)	
Intravenous	8 (2.4)	6 (1.8)	
Erythropoiesis-stimulating agent	58 (17.3)	28 (8.3)	< 0.001
B12 injections	7 (2.1)	6 (1.8)	0.77^b^

^a^mean (s.d.)

^b^McNemar’s exact test due to low frequency of discordant pairs

Use of prophylactic medications (proton pump inhibitor, trimethoprim-sulfamethoxazole and valganciclovir) reduced from 6 to 12 months, consistent with practice guidelines. Use of renin-angiotensin system inhibitors increased slightly at 12 months but remained < 20% overall. A quarter of patients received antiplatelet agents, mostly with aspirin as a single agent. Less than 5% were on anticoagulants, which were similar at both times. There was low use of supplemental iron and B12 injections were limited to those with documented low B12.

### Laboratory characteristics

Results are summarised in Table 3. Haemoglobin and haematocrit were higher at 12 months than 6 months. This was associated with a small reduction in mean corpuscular volume associated with a lower ferritin level, on average. Serum iron, iron binding capacity and transferrin saturation were similar at both time points. However, the proportion of patients with actual laboratory defined low ferritin levels was not different. On the other hand, there was a smaller proportion of patients with transferrin saturation < 10% at 12 months compared to 6 months.

**Table 3:** Comparison of laboratory parameters at 6 and 12 months

Parameter	6 months (n)	12 months (n)	*P* value^b^ (n)
Haemoglobin (g/L)	127 ± 18 (336)	133 ± 18 (336)	< 0.001 (336)
Haematocrit (%)	39.2 ± 0.1 (336)	40.7 ± 0.1 (336)	< 0.001 (336)
Percentage with haematocrit > 0.51	1.5 (336)	3.0 (336)	0.10 (336)
Mean cell volume(fL)	89 ± 7 (336)	87 ± 7 (336)	< 0.001 (336)
White cell count (× 10^9^/L)	6.9 ± 2.8 (336)	7.4 ± 2.6 (336)	0.002 (336)
^a^Serum iron (μmol/L)	13 (9–18) (248)	13 (10–17) (295)	0.44^c^ (225)
Iron binding capacity (μmol/L)	60.5 ± 10.6 (229)	62.1 ± 11.1 (274)	0.06 (209)
^a^Transferrin saturation (%)	23 (15–30) (248)	22 (16–30) (292)	0.48^c^ (225)
Percentage with TSAT < 20%	40.7 (248)	37.7 (292)	0.32^d^ (225)
Percentage with TSAT < 10%	12.9 (248)	7.9 (292)	0.02^d^ (225)
^a^Ferritin (μg/L)	95 (37–269) (248)	75 (31–193) (294)	< 0.001^c^ (227)
Percentage with ferritin < 20 μg/L	12.5 (248)	13.6 (294)	0.37^d^ (227)
Transferrin (g/L)	2.43 ± 0.43 (247)	2.48 ± 0.46 (291)	0.11 (225)
^a^Serum B_12_ (pmol/L)	299 (217–442) (222)	268 (199–380) (284)	0.002^c^ (197)
Percentage with B_12_ < 140 pmol/L	7.7 (222)	6.7 (284)	0.56^d^ (197)
Percentage with serum folate < 10 or red cell folate < 800 nmol/L	2.3 (222)	2.2 (278)	0.41^d^ (193)
Creatinine (μmol/L)	125 ± 44 (336)	126 ± 52 (336)	0.29 (336)
CKD-EPI eGFR (ml/min/m^2^)	56.1 ± 18.5 (336)	56.3 ± 19.0 (336)	0.77 (336)
^a^PTH (pmol/L)	9.8 (6.9–15.5) (244)	9.5 (6.6–15.1) (299)	0.022^c^ (232)
Percentage with PTH > 20 pmol/L	16.4 (244)	14.4 (299)	0.13 (232)
Proteinuria (%)	23.8 (336)	26.2 (336)	0.22^d^ (336)

Results are mean ± s.d. or ^a^median (interquartile range). ^b^Significance test is based on paired data, which is paired t-test unless specified as ^c^Wilcoxon signed rank test or ^d^McNemar’s test

Overall, B12 levels were lower at 12 months than 6 months but the proportion with actual deficiency was similar. Folate deficiency was rare. Overall, kidney function remained stable between 6 and 12 months with a mean eGFR of 56 ml/min/m^2^. PTH levels were lower at 12 months but there was a similar proportion with PTH levels greater than 3 times the upper limit of normal. The proportion of patients with proteinuria was similar at both time points.

### Moderate-severe anaemia and ESA use

We confirmed that all patients receiving ESA had a haemoglobin less than 100 g/L at initiation. The proportion of patients using ESAs dropped from 17.3% at 6 months to 8.3% at 12 months. This difference of 8.9% (95% CI: 4.5–13.4%) was statistically significant (*X*^2^ = 16.67, df = 1, *P* < 0.001). The prevalence of anaemia based on WHO criteria is shown in Table 4. There is very strong evidence that the proportion of patients with moderate-severe anaemia or ESA use was lower at 12 months than at 6 months (*X*^2^ = 23.68, df = 1, P < 0.001). The difference in the proportion of anaemic patients was 12.2% (95% CI: 7.2–17.2%).

**Table 4:** Prevalence of anaemia at 6 and 12 months (*n* = 336)

	6 months	12 months
Original WHO categories		
No anaemia	54.4%	67.3%
Mild anaemia	28.0%	22.9%
Moderate-severe anaemia	17.6%	9.8%
Study categories (inclusive of ESA use)		
No/mild anaemia	72.6%	84.8%
Moderate-severe anaemia	27.4%	15.2%

WHO criteria (mild anaemia males: 110–129 g/L, females: 110–119; moderate: < 110, severe: < 80)

### Factors associated with moderate-severe anaemia

The results of univariate analysis are shown in Additional file 1: Table S1. In multivariable logistic regression analysis, the significant factors associated with moderate-severe anaemia at 6 months were: female gender, allograft function, transferrin saturation < 10%, recent treatment with IVIG and proteinuria (Table 5). Every 5 ml/min/m^2^ increase in eGFR was associated with 19% lower odds of having moderate-severe anaemia at 6 months. The c-statistic for this model was 0.79.

**Table 5:** Risk factors for moderate-severe anaemia in multivariable modelling (*n* = 336)

	Odds ratio	95% C.I.	*P* value
6 months			
eGFR/5 (ml/min/m^2^)	0.81	0.74–0.88	< 0.001
Female sex	4.26	2.41–7.55	< 0.001
Recent intravenous immunoglobulin^a^	2.28	1.12–4.63	0.023
Transferrin saturation < 10%	3.87	1.69–8.90	0.001
Proteinuria	1.95	1.05–3.60	0.035
12 months			
eGFR/5 (ml/min/m^2^)	0.80	0.71–0.89	< 0.001
Female sex	3.12	1.46–6.66	0.003
Recent acute rejection^a^	3.09	1.27–7.53	0.013
Recent infection^b^	2.80	1.21–6.51	0.016
Transferrin saturation < 10%	3.45	1.11–10.76	0.033
Proteinuria	2.69	1.29–5.61	0.008

^a^within the last 3 months

^b^within the last 4 weeks

The significant factors associated with moderate-severe anaemia at 12 months were: female gender, allograft function, recent rejection, recent infection, transferrin saturation < 10% and proteinuria (Table 5). Every 5 ml/min/m^2^ increase in eGFR was associated with 20% lower odds of having moderate-severe anaemia at 12 months. The c-statistic for this model was 0.86.

There was a linear trend in the association between urine protein-creatinine ratio and moderate-severe anaemia (Table 6) at 6 months (*X*^2^ = 4.30, df = 1, *P* = 0.038) and 12 months (*X*^2^ = 4.43, df = 1, *P* = 0.035), after adjusting for the relevant covariates. However, including proteinuria as an ordinal rather than binary variable in the multivariable models resulted in nearly identical coefficients and confidence intervals for the covariates. The c-statistics were also unchanged. Thus, using proteinuria as a binary variable in the multivariable models is parsimonious and did not result in loss of model discrimination.

**Table 6:** Logistic regression showing association of different levels of proteinuria with moderate-severe anaemia (*n* = 336)

Urine protein/creatinine (g/mmol)	Odds ratio	95% C.I.	*P* value
6 months^a^			
≤ 0.03	1.00	reference	0.052
> 0.03 to ≤0.1	1.69	0.88–3.26	
> 0.1	4.00	1.09–14.6	
12 months^b^			
≤ 0.03	1.00	reference	0.023
> 0.03 to ≤0.1	2.43	1.09–5.40	
> 0.1	3.70	1.18–11.6	

Note: The odds ratios and 95% confidence intervals for the covariates were nearly identical to the multivariable models in Table 5 (with proteinuria as a binary variable)

^a^adjusted for eGFR, sex, intravenous immunoglobulin use, transferrin saturation < 10%

^b^adjusted for eGFR, sex, acute rejection, recent infection, transferrin saturation < 10%

To determine the impact of including ESA use in the definition of moderate-severe anaemia, we performed a comparison logistic regression analysis with moderate-severe anaemia defined by the original WHO criteria (Additional file 2: Table S2). In this comparison analysis, we excluded patients using ESAs who did not have moderate-severe anaemia (patients included purely on ESA criteria independent of WHO criteria; *n* = 33 at 6 months, *n* = 18 at 12 months). In the comparison analysis, recent rejection at 12 months was not significantly associated with the outcome (odds ratio, 1.07 [95% CI: 0.27–4.30], *P* = 0.92), after allowing for the other covariates. There was also little evidence that proteinuria was associated with the outcome at 6 months (odds ratio, 2.00 [95% CI: 0.97–4.18], *P* = 0.06). The association of the other factors with moderate-severe anaemia remain significant.

### Blood transfusions

**Fig. 2:**
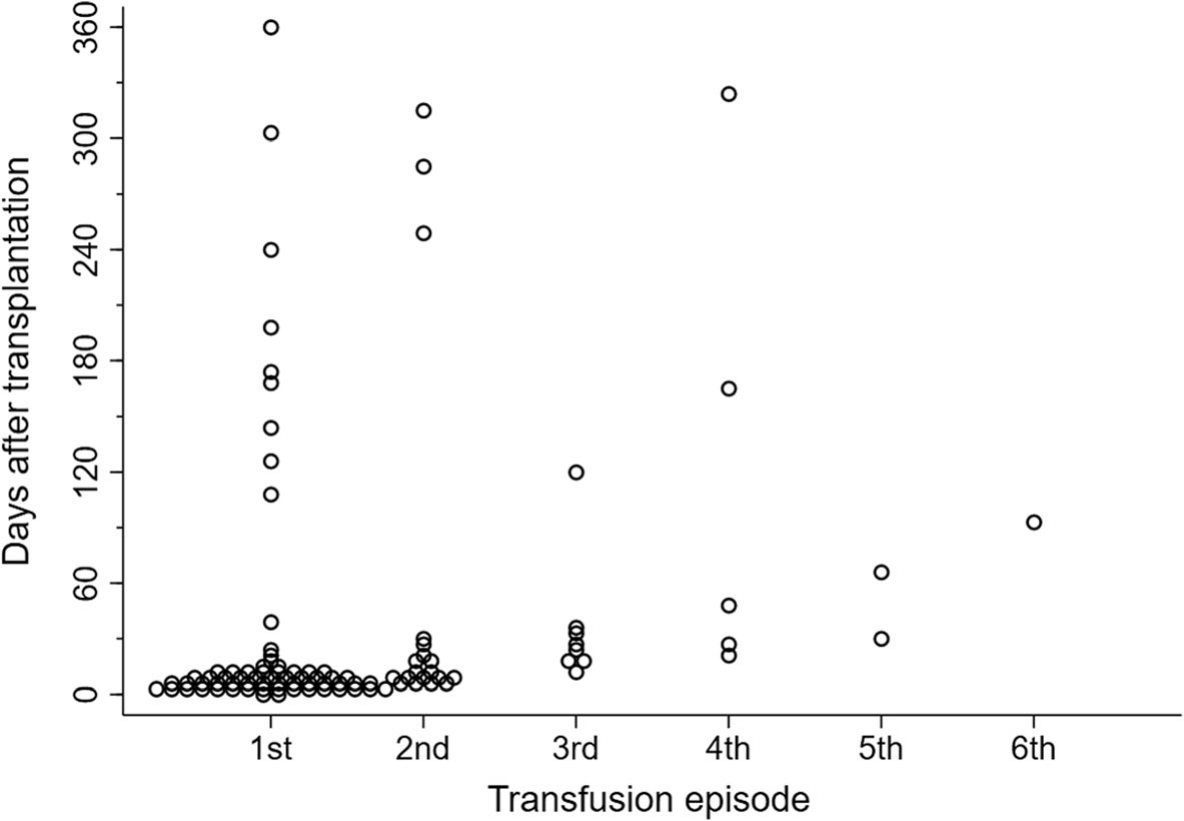
Plot showing timing of transfusions in 66 patients who received at least one blood transfusion after transplantation. Each circle represents a transfusion episode. Time after transplantation is shown on the y-axis and the transfusion episode number on the x-axis. Most transfusions occurred in the early post-transplant period and the majority of patients only had one or two transfusion episodes

A total of 66/336 (19.6%) patients had at least one transfusion episode within 12 months of transplantation, excluding intra-operative transfusions. Of these, 47/66 (71%) had only one transfusion episode. The mean ± s.d. number of transfusions per episode was 1.4 ± 0.7 units (median number of transfusions per episode = 1 unit, interquartile range = 1 unit). The timing of all transfusion episodes in relation to time after transplantation is shown in Fig. 2.

Half of all transfusions occurred in the immediate post-operative period (within 10 days of transplantation) and 75% occurred within 25 days of transplantation. There were 13/336 (3.9%) patients who had at least one transfusion episode after 3 months. Of these 10/13 were already classified as having moderate-severe anaemia at 6 months. There were 4/336 (1.2%) patients with at least one transfusion episode after 9 months. All except one were already classified as having moderate-severe anaemia. A sensitivity analysis was conducted assuming a worst-case scenario that these four patients were misclassified due to a transfusion within the last 3 months (data not shown). There was no statistically meaningful change in the final multivariable model and the c-statistic remained the same.

## Discussion

The prevalence of anaemia in this study of 32.7% at 12 months post-transplant is consistent with estimates reported in the literature. However, a large proportion of these patients only had mild anaemia. For further discussion, we refer to the WHO-defined moderate-severe anaemia (haemoglobin < 110 g/L) in this study simply as “anaemia”. We did not examine anaemia risk in the immediate post-surgical period as this is mostly related to surgical issues, transient haemodilution due to fluid loading and the significant volume of early phlebotomies [12]. These risk factors may not necessarily be modifiable so our focus was on anaemia beyond 3 months. We noted that the prevalence of anaemia declined between 6 and 12 months, which is consistent with other studies [8].

There was also a low prevalence of polycythaemia (haematocrit > 0.51) of 1.5% at 6 months and 3.0% at 12 months. These patients were exclusively male. Of these, 2/5 and 3/10 had PKD at 6 and 12 months, respectively. Our prevalence of polycythaemia is relatively low compared to previous data, and may certainly reflect changes in transplantation practice, which was as high as 19% in the mid-1990’s and around 8% in the early 2000’s [13]. One of these differences may be the proportions of patients with PKD in the transplant cohort, increasing use of renin-angiotensin system inhibitors or impact of MMF use.

Among patient factors, female sex has been associated with moderate-severe anaemia at both time points. This sex association is noted in a number of other studies as well [14–16]. It is postulated that this may partly be due to an increase in irregular menses and menorrhagia after kidney transplantation compared to prior [17]. This abnormal bleeding may be associated with changes to the hormonal profile post-transplantation [18]. However, we did not have data on menopausal status to address this hypothesis. Recipient age showed no association with moderate-severe anaemia even when stratified by sex (data not shown). We found no association between donor type and delayed graft function with anaemia. Diabetic status was associated with anaemia in univariate analysis at 12 months but not multivariate analysis. The highest risk group may be those with new-onset diabetes after transplantation.

Poor graft function is a very consistent correlate with anaemia across many transplant centres and studies [19, 20]. Indeed, this was confirmed in our study as well. A low eGFR (estimated by CKD-EPI equation) was associated with anaemia, even after adjusting for rejection and infection. A similar finding was noted if serum creatinine was used instead of eGFR (data not shown). The increased odds may be related to the inherent quality of the donor kidney and some studies have found an association between donor age and anaemia [20–22]. With the increasing use of extended criteria donors, this may be an area of concern which requires further study. Serum PTH was associated with anaemia in univariate but not multivariate analysis, presumably a reflection of its association with underlying allograft function.

In our study, proteinuria was associated with anaemia even when adjusted for renal function, and the odds of anaemia increases with higher levels of proteinuria, particularly at 12 months. A retrospective study by Bonofiglio et al. noted an association between anaemia at 1 year and 24-h proteinuria at 6 months in their multivariable model [23]. It is unlikely that proteinuria itself causes anaemia but may be a surrogate for other phenomena. There are several postulated mechanisms on how patients with nephrotic syndrome are at increased risk of anaemia. As reviewed by Iorember et al., this may involve increased urinary losses of iron, B12-transcobolamin, caeruloplasmin (secondary copper deficiency) and erythropoietin [24]. However, whether these mechanisms are involved in patients with sub-nephrotic proteinuria or transplantation is unclear.

In our study, we examined the haematinic profile. Although iron deficiency is common, there is much debate surrounding the definitions and whether the parameters used in the general population are applicable in the post-transplant setting. On average, we noted that serum ferritin and B12 levels were lower at 12 months compared to 6 months. However, the prevalence of laboratory defined deficiency based on standard cut-offs were not different. Serum ferritin was unhelpful and paradoxical, with anaemic patients having higher ferritin than non-anaemic patients (316 ± 359 μg/L vs. 125 ± 152 μg/L, t_292_ = − 5.91, *P* < 0.001). Serum ferritin levels also showed a positive association with anaemia. This may relate to the inflammatory condition and functional iron deficiency. Thus, the ideal level to define deficiency is a little unclear. Similarly, we found that a serum transferrin saturation below 20% as a standard cut-off was not associated with anaemia but a threshold of 10% did in both univariate and multivariate models. It was difficult to analyse folate levels as a continuous variable due to the change in laboratory reporting from red cell folate to serum folate during the study period. Nonetheless, folate deficiency is uncommon (2% prevalence) with no demonstrable association with anaemia.

It has been previously suggested that a poor response to ESAs pre-transplant was a predictor of post-transplant anaemia [25]. However, we did not collect data on pre-transplant haematinic and haematological parameters. Given that ESA and iron supplementation are usually ceased at the time of transplantation, it is unclear how relevant these baseline values are at 6 months. Furthermore, it has been shown that iron deficiency can develop by 6 months in over half of patients who were iron-replete prior to transplantation [26]. There may also be an association between the malnutrition-inflammation score and post-transplant anaemia [19]. These factors could not be assessed in our study.

The use of azathioprine and MMF as anti-proliferative agents has been associated with anaemia. In our centre, MMF use is almost universal within the first 12 months of transplantation so we were unable to compare these two agents. The proportion of patients on daily MMF doses < 1.5 g was higher at 12 months but we could not detect a statistically significant association with the lower dose and anaemia using logistic regression. However, MMF dose could have been transiently reduced on occasions due to incidental leukopaenia and this may have reduced our ability to detect an association between MMF dose and anaemia. The use of mammalian target of rapamycin inhibitors is also associated with anaemia [27, 28]. In our centre, mammalian target of rapamycin inhibitor use was around 3% in the first 12 months and no association with anaemia could be detected.

In the general population, renin-angiotensin system inhibitors are associated with a 50–60% higher risk of anaemia [29]. In our study, there was no obvious effect of renin-angiotensin system inhibitors on anaemia. In the SMAhRT study of telmisartan versus placebo followed for a mean duration of 15 months, use of telmisartan did not worsen anaemia [30]. Nonetheless, it is unclear if the use of renin-angiotensin system inhibitors has contributed to the low prevalence of polycythaemia as previously mentioned. The use of proton-pump inhibitors has also been linked to poor iron absorption, contributing to iron-deficiency in some patients in the general population. Proton-pump inhibitors are routinely prescribed after transplantation but variably maintained and no information is available regarding its impact on iron status in the transplant population. We noted that patients on proton-pump inhibitors had a lower transferrin saturation than those who did not, at 12 months (27.5% ± 12.3% vs. 23.0 ± 11.0%, t_290_ = 2.32, *P* = 0.021). There was a suggestion of an association between proton-pump inhibitor use and anaemia on univariate analysis which did not reach statistical significance (*P* = 0.07). If may be useful to explore this potential association in future studies. We did not find an association between trimethoprim-sulfamethoxazole or valganciclovir use with anaemia. Finally, the prevalence of ESA use of 8.1% in this study is comparable to previous reports of 5–11% [14, 20].

Recent rejection was associated with anaemia at 6 and 12 months in univariate analysis. It remained significant at 12 months with multivariate analysis but not in the comparison analysis using WHO criteria for anaemia and excluding ESA-treated, non-anaemic patients. The mechanism of rejection mediated anaemia is likely multifactorial, with both reduced erythropoietin production and inflammation-related erythropoietin resistance at play. However, we also noted that recent IVIG treatment for antibody-mediated rejection was associated with anaemia at 6 months. There is a theoretical risk of high dose (2 g/kg) IVIG precipitating haemolysis in transplant patients [31]. It was proposed that particular blood groups (A, B or AB) and IVIG preparations may be more likely to be associated with haemolysis. In non-transplant patients, data from neurological studies also showed recurrent high-dose IVIG use was associated with reduction in haematocrit or haemoglobin, and that biochemical evidence of haemolysis may be present even if an overt haemolytic syndrome was not evident [32, 33].

In our study, a clinically evident recent infection within the last 4 weeks was associated with anaemia. The majority of these were urinary tract and respiratory infection. Infections possibly cause derangements in iron utilization and erythropoietin resistance. Of further note, opportunistic infections such as cytomegalovirus, Epstein-Barr virus, BK virus and parvovirus B19 can cause direct bone marrow suppression. In our study, cytomegalovirus infection within the last 3 months was associated with anaemia at 6 months in univariate analysis but not in the multivariate model. One possible confounder of the association between anaemia and rejection or infection is the increased burden of diagnostic phlebotomy during acute management. This is difficult to tease out as data on frequency and volume of blood loss was not estimated.

### Strengths and limitations

The strengths of this study include the full evaluation of ESA use and consideration of the potential impact of ESA use on prevalence of anaemia. Anaemia prevalence can be underestimated if patients are rescued from anaemia by ESAs. We also incorporated an audit of blood transfusions received by patients to determine transfusion requirements and potential impact of transfusions on anaemia prevalence. This study also evaluated two time points to determine if the factors associated with anaemia evolved over time, rather than assuming that any factors associated with anaemia remain stable between 6 and 12 months.

Including ESA use in the definition of anaemia can also be a limitation by introducing complexity or bias into the analysis. We attempted to address this by performing a comparison analysis using WHO criteria alone to define the outcome. Ultimately, a prospective study collecting incident data would be needed to confirm these associations.

This was a single-centre study and the cross-sectional design means that the results cannot be used for causal inference. A longitudinal study would be useful to confirm the identified factors associated with anaemia as specific risk factors. It was also not designed to look at outcomes such as graft and patient survival.

Data on the use of oral iron supplementation and multivitamins may not be reliable as they were not systematically recorded although data on iron infusions were robust as they were organised through our infusion centre. We also had missing data on haematinics, particularly at 6 months. As mentioned in the methods section, this may be related to true missing values (test not performed) or related to timing. Although we used multiple imputation, the missing data could introduce some bias into the results if they were not truly *missing at random*.

In terms of generalisability, we would caution against generalising these results to kidney transplant cohorts with significant mammalian target of rapamycin inhibitor use in the first 12 months post-transplantation. Given the significant proportion of pancreas-kidney transplant recipients excluded from the study due to lack of clinical data, a similar caution applies to pancreas-kidney transplant cohorts. Our models should be validated in cohorts with more complete data from such transplant recipients.

### Implications for practice

We have learned that anaemia prevalence can be underestimated when ESA use is not considered. Transplant centres monitoring anaemia prevalence should take this into account. The use of IVIG should be considered in the differential diagnosis of anaemia in kidney transplant patients, which may otherwise appear unexplained. A transferrin saturation below 10% should be a prompt to consider iron supplementation even if serum ferritin is within the normal range.

## Conclusions

Post-transplant anaemia remains prevalent even in the modern transplant era. Female gender, allograft function, rejection and infection are associated with moderate-severe anaemia. Iron studies are difficult to interpret in the first post-transplant year but a transferrin saturation less than 10% may be a useful marker of increased risk. The role of proton-pump inhibitors, proteinuria and IVIG use in the development of anaemia requires further study. We also recommend that future studies include a quantitative analysis of protein excretion to confirm if there is a linear increase in the risk of anaemia with increasing levels of proteinuria.

## Additional files

Additional file 1: **Table S1.** Univariate logistic regression analysis. The supplementary table shows the results of univariate analysis with the unadjusted odds ratio, 95% confidence intervals and significance values. It also shows the number of observations with the outcome of interest and the total number of observations where data is available. (DOCX 35 kb)

Additional file 2: **Table S2.** Factors associated with WHO criteria defined moderate-severe anaemia in multivariable modelling. This analysis excludes patients who only met ESA criteria independent of WHO criteria for the definition of moderate-severe anaemia. It demonstrates that the factor “recent acute rejection” was no longer significantly associated with moderate-severe anaemia after allowing for the other covariates. (DOCX 17 kb)

## Abbreviations

CKD: Chronic kidney disease
eGFR: Estimated glomerular filtration rate using CKD-EPI
ESA: Erythropoiesis-stimulating agent
IVIG: Intravenous immunoglobulin
MMF: Mycophenolate mofetil
PKD: Polycystic kidney disease
PTH: Parathyroid hormone
WHO: World Health Organization
