# Supplementary material for: Factors associated with anaemia in kidney transplant recipients in the first year after transplantation: a cross-sectional study
Source: BMC Nephrol. 2018 Oct 5;19:252. doi: 10.1186/s12882-018-1054-7 (PMC6173839; doi:10.1186/s12882-018-1054-7)
Supplement: Supplementary file 1 — Table S1. Univariate logistic regression analysis. The supplementary table shows the results of univariate analysis with the unadjusted odds ratio, 95% confidence intervals and significance values. It also shows the number of observations with the outcome of interest and the total number of observations where data is available. (DOCX 35 kb) [file 12882_2018_1054_MOESM1_ESM.docx]

**Table S1: Univariate logistic regression analysis**

| **Variable** | **6 months** | | |  | **12 months** | | |
| --- | --- | --- | --- | --- | --- | --- | --- |
|  | **n/total** | **OR (95% CI)** | **P** |  | **n/total** | **OR (95% CI)** | **P** |
| Age* | -/336 | 1.00 (0.99-1.02) | 0.63 |  | -/336 | 1.01 (0.98-1.03) | 0.49 |
| Female gender | 49/336 | 3.41 (1.91-6.09) | <0.001 |  | 49/336 | 3.22 (1.54-6.74) | 0.002 |
| Donor  Brain death  Cardiac death  Living  ABO incompatible | 176/336  63/336  82/336  15/336 | 1.00 (reference)  1.00 (0.54-1.88)  0.65 (0.35-1.21)  0.36 (0.08-1.64) | 0.27 |  | 176/336  63/336  82/336  15/336 | 1.00 (reference)  1.43 (0.69-2.99)  0.77 (0.35-1.67)  0.39 (0.05-3.12) | 0.40 |
| Delayed graft function | 83/336 | 1.62 (0.95-2.76) | 0.08 |  | 83/336 | 1.49 (0.78-2.85) | 0.23 |
| Kidney-pancreas transplant | 23/336 | 1.17 (0.47-2.95) | 0.73 |  | 23/336 | 1.19 (0.39-3.66) | 0.76 |
| Polycystic kidney disease | 37/336 | 0.98 (0.45-2.11) | 0.96 |  | 37/336 | 0.86 (0.32-2.32) | 0.76 |
| Diabetes  Type 1  Type 2  New-onset after transplant | 39/336  71/336  17/336 | 1.49 (0.73-3.07)  0.66 (0.34-1.27)  1.87 (0.68-5.14) | 0.17 |  | 39/336  71/336  17/336 | 2.09 (0.86-5.08)  1.81 (0.86-3.80)  5.66 (1.96-16.3) | 0.012 |
| Risk factors for GI bleeding | 59/336 | 0.98 (0.52-1.85) | 0.96 |  | 59/336 | 0.85 (0.38-1.92) | 0.70 |
| Recent overt bleeding | 17/336 | 2.49 (0.93-6.66) | 0.07 |  | 11/336 | 2.16 (0.55-8.45) | 0.27 |
| Recent rejection episode | 47/336 | 2.23 (1.18-4.22) | 0.013 |  | 38/336 | 3.07 (1.43-6.57) | 0.004 |
| Recent infection | 70/336 | 2.28 (1.31-3.96) | 0.004 |  | 46/336 | 3.89 (1.93-7.84) | <0.001 |
| Recent CMV | 13/336 | 3.27 (1.07-9.99) | 0.038 |  | 13/336 | 2.61 (0.77-8.82) | 0.12 |
| Recent BKV | 51/336 | 0.52 (0.24-1.12) | 0.10 |  | 48/336 | 0.61 (0.23-1.63) | 0.33 |
| MMF dose <1.5g/day | 70/336 | 1.52 (0.86-2.67) | 0.15 |  | 107/336 | 1.05 (0.51-2.18) | 0.14 |
| Prednisolone dose* | -/336 | 1.06 (0.97-1.15) | 0.22 |  | -/336 | 1.19 (1.01-1.40) | 0.034 |
| Recent IVIG | 49/336 | 2.53 (1.35-4.71) | 0.004 |  | 28/336 | 3.62 (1.56-8.38) | 0.003 |
| Recent plasma exchange | 18/336 | 1.02 (0.35-2.95) | 0.97 |  | 8/336 | 3.50 (0.81-15.1) | 0.10 |
| Proton pump inhibitor use | 301/336 | 1.31 (0.87-2.99) | 0.53 |  | 287/336 | 3.08 (0.92-10.3) | 0.07 |
| Cotrimoxazole use | 301/336 | 0.69 (0.33-1.46) | 0.34 |  | 256/336 | 0.90 (0.45-1.79) | 0.76 |
| Valganciclovir use | 180/336 | 1.42 (0.87-2.30) | 0.16 |  | 51/336 | 1.68 (0.80-3.55) | 0.17 |
| RAS blocker use | 49/336 | 0.95 (0.48-1.89) | 0.89 |  | 60/336 | 0.98 (0.45-2.15) | 0.97 |
| Antiplatelet/anticoagulants | 97/336 | 0.61 (0.35-1.06) | 0.08 |  | 98/336 | 0.81 (0.41-1.59) | 0.53 |
| Transferrin saturation <20% | 101/248 | 1.42 (0.82-2.45) | 0.21 |  | 110/292 | 1.29 (0.66-2.50) | 0.45 |
| Transferrin saturation <10% | 32/248 | 3.09 (1.45-6.58) | 0.004 |  | 23/292 | 2.93 (1.12-7.61) | 0.028 |
| Ferritin <20 ug/L | 31/248 | 0.94 (0.41-2.14) | 0.88 |  | 40/294 | 0.37 (0.14-0.98) | 0.045 |
| B12 <140 pmol/L | 17/222 | 2.14 (0.79-5.83) | 0.13 |  | 19/284 | 1.09 (0.30-3.90) | 0.90 |
| Red cell folate <800 nmol/L or serum folate <10 nmol/L | 5/222 | 0.55 (0.06-4.99) | 0.59 |  | 6/278 | 1.13 (0.13-9.89) | 0.91 |
| CKD-EPI eGFR* | -/336 | 0.96 (0.95-0.98) | <0.001 |  | -/336 | 0.95 (0.93-0.97) | <0.001 |
| PTH level* | -/244 | 1.01 (0.98-1.03) | 0.61 |  | -/299 | 1.04 (1.01-1.06) | 0.008 |
| PTH >20 pmol/L | 40/244 | 1.59 (0.78-3.22) | 0.20 |  | 43/299 | 4.04 (1.94-8.41) | <0.001 |
| Proteinuria | 80/336 | 2.34 (1.38-3.99) | 0.002 |  | 88/336 | 4.13 (2.23-7.67) | <0.001 |

n/total = frequency of event/total observations available. *continuous variables.
