# Supplementary material for: Factors associated with anaemia in kidney transplant recipients in the first year after transplantation: a cross-sectional study
Source: BMC Nephrol. 2018 Oct 5;19:252. doi: 10.1186/s12882-018-1054-7 (PMC6173839; doi:10.1186/s12882-018-1054-7)
Supplement: Supplementary file 2 — Table S2. Factors associated with WHO criteria defined moderate-severe anaemia in multivariable modelling. This analysis excludes patients who only met ESA criteria independent of WHO criteria for the definition of moderate-severe anaemia. It demonstrates that the factor “recent acute rejection” was no longer significantly associated with moderate-severe anaemia after allowing for the other covariates. (DOCX 17 kb) [file 12882_2018_1054_MOESM2_ESM.docx]

**Table S2: Factors associated with WHO criteria defined moderate-severe anaemia in multivariable modelling (n=303 at 6 months, n=318 at 12 months)^†^**

|  | **Odds ratio** | **95% C.I.** | **P value** |
| --- | --- | --- | --- |
| **6 months** |  |  |  |
| eGFR/5 (ml/min/m^2^) | 0.79 | 0.71 – 0.88 | <0.001 |
| Female gender | 5.35 | 2.68 – 10.7 | <0.001 |
| Recent intravenous immunoglobulin* | 2.32 | 1.02 – 5.29 | 0.045 |
| Transferrin saturation <10% | 4.72 | 1.80 – 12.4 | 0.002 |
| Proteinuria | 2.00 | 0.97– 4.18 | 0.065 |
| **12 months** |  |  |  |
| eGFR/5 (ml/min/m^2^) | 0.79 | 0.70 – 0.91 | 0.001 |
| Female gender | 6.15 | 2.21 – 17.2 | 0.001 |
| Recent acute rejection* | 1.07 | 0.27 – 4.30 | 0.92^‡^ |
| Recent infection** | 4.54 | 1.79 – 11.5 | 0.001 |
| Transferrin saturation <10% | 8.42 | 2.25 – 31.4 | 0.002 |
| Proteinuria | 3.49 | 1.38 – 8.83 | 0.008 |

^†^Patients with a haemoglobin ≥110 g/L while receiving erythropoiesis-stimulating agents excluded.

^‡^Model remains stable when this factor is dropped from the multivariable analysis.

*within the last 3 months

**within the last 4 weeks
